# Mutational Landscape of Primary Hyperoxaluria in Morocco: Update and Implications for Diagnosis

**DOI:** 10.3390/genes17091056

**Published:** 2026-08-31

**Authors:** Ourayna Batta, Yasmina Rahmuni, Jaber Lyahyai, Zineb Rchiad, Yassamine Doubaj, Abdelaziz Sefiani, Imane Cherkaoui Jaouad

**Affiliations:** 1 Research Team in Genomics and Molecular Epidemiology of Genetic Diseases, Genomics of Human Pathologies Center, Faculty of Medicine and Pharmacy of Rabat, University Mohammed V, Rabat 10100, Morocco; battaourayna@gmail.com (O.B.); rahmuniyasmina.24@gmail.com (Y.R.); jaber.lyahyai@gmail.com (J.L.); sefianigen@hotmail.com (A.S.); 2 Department of Medical Genetics, National Institute of Health, Rabat 10090, Morocco; y1doubaj@gmail.com; 3 Genomics Platform, Biosciences CoreLab, Mohammed VI Polytechnic University (UM6P), Ben Guerir 43150, Morocco; zineb.rchiad@um6p.ma; 4 Unity of Medical Genetics, Children’s Hospital, Ibn Sina University Hospital Center, Faculty of Medicine and Pharmacy, Mohammed V University of Rabat, Rabat 10100, Morocco

**Keywords:** primary hyperoxaluria, PH1, NGS, customized panel, WES

## Abstract

Introduction: Primary hyperoxaluria (PH) is a rare autosomal recessive disease characterized by an excess of oxalate, which results in nephrolithiasis, nephrocalcinosis, and ultimately, renal failure and systemic oxalosis. There are 3 forms of PH, named types 1, 2, and 3, caused by variants in the *AGXT*, *GRHPR*, and *HOGA1* genes, respectively. In Morocco, where consanguinity is common, recurrent variants, mainly affecting the *AGXT* gene, have been documented; however, the overall molecular profile of PH remains poorly characterized. We aim here to describe the mutational landscape of PH in Morocco and to provide an accurate diagnostic approach. Methods: We analyzed 132 patients referred over 10 years for PH using a stepwise strategy. A step 1 test involved Sanger sequencing of exon 7 of the *AGXT* gene, followed by sequencing of exons 1, 2, and 10 of the *AGXT* gene. The step 2 analysis consists of a customized gene panel targeting the three PH-associated genes. Unresolved cases underwent whole-exome sequencing as a third step. Results: First-line Sanger sequencing identified biallelic pathogenic or likely pathogenic AGXT variants in 95 of 132 patients suspected of PH, corresponding to a diagnostic yield of 72%. Additional molecular diagnoses obtained through targeted PH panel sequencing and WES increased the final cumulative diagnostic yield to 78%. Twelve distinct variants were characterized by Sanger sequencing, with the c.731T>C in exon 7 of the *AGXT* gene, emerging as the most prevalent variant, supporting its founder effect in this population. Other variants were predominantly located in exons 7, 10, 2, and 1, highlighting mutation hotspots in this population. In unresolved cases, next-generation sequencing (NGS), including targeted gene panels and exome sequencing, uncovered additional pathogenic variants in the *AGXT* gene and variants in other genes associated with PH-like phenotypes. Conclusions: These results highlight the need for a simplified, exon-focused diagnostic strategy, particularly in resource-limited settings like Morocco.

## 1. Introduction

Primary hyperoxaluria (PH) is a group of autosomal recessive disorders that affect glyoxylate metabolism in the liver, leading to excessive production of oxalate, a final product of metabolism that is almost exclusively excreted by the kidneys. The increased urinary excretion of oxalate leads to calcium oxalate precipitation, causing nephrocalcinosis and recurrent kidney stones. Oxalate toxicity causes tubulointerstitial inflammation and renal failure, affecting more than 70% of patients with primary hyperoxaluria [1]. Once hepatic oxalate production exceeds the renal excretory capacity, systemic oxalate accumulation increases, with oxalate depositing in various tissues, such as bones, the heart, blood vessels, nerves, and eyes, which can result in severe, potentially life-threatening multi-organ damage.

Three distinct subtypes of primary hyperoxaluria (PH) are currently recognized. Primary hyperoxaluria type 1 (PH1; MIM 259900), the most severe form, is an autosomal recessive disorder caused by variants in the *AGXT* gene, which encodes the hepatic enzyme alanine–glyoxylate aminotransferase (AGT). The less common subtypes, PH2 and PH3, are associated with variants in the *GRHPR* and *HOGA1* genes, respectively [2].

In PH1, deficiency in the hepatic enzyme AGT, which catalyzes the conversion of glyoxylate to glycine, leads to abnormal accumulation of glyoxylate, which is subsequently converted to oxalate leading to calcium oxalate crystal deposition. This pathologic process causes nephrolithiasis and nephrocalcinosis, with progression to end-stage renal disease and systemic oxalosis in advanced cases.

The prevalence of PH1 remains largely underestimated. In Europe, the prevalence is estimated at 1–3 cases per million, whereas this disease appears to be more prevalent in regions with high rates of consanguinity such as the Middle East and North Africa.

In Morocco, PH1 appears to be relatively frequent, with an estimated prevalence ranging from 1 in 7267 to 1 in 6264 individuals [3]. The elevated prevalence of PH1 in the Moroccan population may be attributed to the high frequency of consanguineous marriages [4].

Although definitive diagnosis of PH1 has traditionally relied on liver biopsy with measurement of hepatic AGT enzyme activity [5], molecular genetic testing has emerged as a simple and noninvasive method for confirming PH1 in index cases. PH1 results from variants in the AGXT gene, which is located on chromosome 2 (2q37.3) and comprises 11 exons, spanning approximately 10 kb [6]. To date, 299 variants implicated in the disease have been documented, with all exons represented, according to the Human Gene Mutation Database (HGMD) (http://www.hgmd.cf.ac.uk) (accessed on 3 May 2026). The molecular investigation of the *AGXT* gene in a previous study demonstrated that the c.731T>C (p.Ile244Thr) was the most frequent mutation [3].

Furthermore, given the phenotypic overlap among the three subtypes, genetic testing is considered essential for their differentiation. In addition, both therapeutic management and responsiveness to vitamin B6 vary according to the patient’s genotype.

Treatment approaches depend on PH subtype: combined liver–kidney transplantation is indicated for PH1, isolated kidney transplantation is mainly considered for PH2, whereas no transplantation is required for PH3 [2]. In addition, the *AGXT* genotype can predict the responsiveness to vitamin B6 supplementation [7]. The present study aims to investigate the mutational landscape in a large cohort of 132 unrelated Moroccan patients with a clinical suspicion of PH. This study would provide a useful road map to establish early diagnosis and appropriate treatment in patients with PH1, which are critical for optimizing patient outcomes.

## 2. Patients and Methods

### 2.1. Patients

Over a period of 10 years, a total of 132 unrelated patients, with a suspected diagnosis of PH, were referred to our center. All patients were from different regions of Morocco; clinical and genealogical data were obtained for each patient.

Among these 132 patients, 74 (56%) were males and 58 (44%) were females. The age at diagnosis ranged from 3 months to 48 years, with an average age of 9 years. The mean age at onset was 7.7 ± 9.8 years, while the median age at onset was 4 years (range: birth–42 years), reflecting marked clinical heterogeneity and the presence of both early and late onset forms. In most cases, symptoms began during early childhood, but the diagnosis was often delayed.

Consanguinity was reported in 84 patients (64%), most commonly due to first-cousin marriage, and a positive family history was documented in 43 patients (32.6%), with at least one affected sibling.

All patients presented with clinical features suggestive of PH: nephrolithiasis (82%), nephrocalcinosis (26.5%), and end-stage renal disease (ESRD) was observed in 40% of patients; among those with available data, the age at renal failure/ESRD ranged from 3 months to 43 years, with a median of 11 years. Among patients for whom urinary oxalate assessment was available (84 patients), elevated urinary oxalate levels were detected in 92.1% of patients. However, biochemical data were incomplete across the cohort, particularly for patients who had already reached ESRD at the time of evaluation.

Because the clinical presentations of the three PH types largely overlap, and the measurement of urinary glycolate to distinguish PH1 from PH2 and PH3 is not available in Morocco, the subtype of PH was not clinically identified prior to genetic testing. Extra-renal or other associated clinical features were reported in five patients, including systemic oxalosis and skeletal involvement.

Informed consent was obtained from each patient, or from a legal guardian in the case of minors, prior to blood sample collection and molecular analysis. This study was approved by the Rabat ethics for biomedical research (Approval Number: CERB-50-24).

### 2.2. DNA Isolation

Genomic DNA was extracted from 200 μL of EDTA-anticoagulated blood using the PureLink™ Genomic DNA Mini Kit (Invitrogen, Thermo Fisher Scientific, Carlsbad, CA, USA) according to the manufacturer’s instructions. DNA concentration was measured by Qubit 3.0 Fluorometer (Invitrogen, Thermo Fisher Scientific, Carlsbad, CA, USA), with the Qubit dsDNA HS (High Sensitivity) Assay Kit (Invitrogen, Thermo Fisher Scientific, Waltham, MA, USA).

### 2.3. Sanger Sequencing

Molecular diagnosis was initially performed by direct Sanger sequencing of exon 7 of the *AGXT* gene. In patients negative for exon 7 variants, exons 1, 2, and 10 were tested, as suggested in a previous Moroccan study [3].

Amplification was checked by electrophoresis on 1% agarose gel. PCR products were then purified using ExoSAP-IT Express reagent ExoSAP-IT Express reagent (Thermo Fisher Scientific Sequencing reactions were performed using a BigDye™ Terminator v3.1 Cycle Sequencing Kit (Applied Biosystems, Thermo Fisher Scientific, Waltham, MA, USA), followed by purification with Sephadex^®^ G-50 method (Sigma-Aldrich, St. Louis, MO, USA). Capillary electrophoresis was carried out on an Applied Biosystems 3500 GeneticAnalyzer (Applied Biosystems, Thermo Fisher Scientific, Waltham, MA, USA). Electropherograms were analyzed and aligned to the *AGXT* reference genomic sequence (GenBank: NM_000030.2) using Mutation Surveyor^®^ software version 5.1.2.

### 2.4. Screening of AGXT, GRHPR, and HOGA1 Genes by Targeted Next-Generation Sequencing

Patients carrying a single heterozygous *AGXT* variant or no pathogenic variant in exons 7, 1, 2, or 10 of the *AGXT* gene were subsequently analyzed by targeted next-generation sequencing. A custom Ion AmpliSeq™ On-Demand Primer Panel (Thermo Fisher Scientific, Waltham, MA, USA) designed using the Ion AmpliSeq™ Designer software version 7.4 (Thermo Fisher Scientific, Waltham, MA, USA), was used to screen the coding regions and exon–intron boundaries of the *AGXT*, *GRHPR*, and *HOGA1* genes associated with the three primary hyperoxaluria subtypes.

A total of 10 ng of genomic DNA per sample was used for target amplification with the Ion AmpliSeq™ On-Demand Primer Panel (Thermo Fisher Scientific, Waltham, MA, USA). Libraries were prepared individually using the Ion AmpliSeq™ Library Kit v2.0 (Thermo Fisher Scientific, Waltham, MA, USA). Amplified products were marked with unique barcoded adapters from the Ion Xpress Barcode Adapters 1–16 Kit. Subsequent amplification and purification steps were performed following the manufacturer’s recommendations. Prepared libraries were quantified with the Qubit™ dsDNA HS Assay Kit to obtain a final concentration of 100 pM per library. Equimolar amounts of barcoded libraries were pooled and processed automatically on the Ion Chef™ System for template preparation using the Ion 510™/520™/530™ Kit-Chef. Sequencing was conducted on the Ion S5™ System using the Ion S5™ Sequencing kit. Raw sequencing data were analyzed using Torrent Suite™ Software v5.0.2. Variant annotation and filtering were performed in Ion Reporter™ Software, version 5.18 (Thermo Fisher Scientific, Waltham, MA, USA).

Variants with a coverage depth below 20× were visually inspected using the Integrative Genomics Viewer (IGV, https://software.broadinstitute.org/software/igv/) (accessed on 14 April 2026). Variants were retained based on the following filtering criteria:

1. Minor allele frequency (MAF) < 0.01 in public population databases, including the 1000 Genomes Project (http://www.internationalgenome.org/), gnomAD (http://gnomad.broadinstitute.org/), and ESP5400;
2. MAF < 0.02 in local resources (in-house database);
3. Compatibility with an autosomal recessive inheritance model;
4. Classification as pathogenic or likely pathogenic according to the American College of Medical Genetics and Genomics (ACMG) guidelines;
5. Whole-exome sequencing.

### 2.5. Library Preparation

Libraries were prepared using the Illumina DNA Prep with Exome 2.5 Enrichment Kit (Illumina, Inc., San Diego, CA, USA), according to the manufacturer’s recommendations. Bead-linked transposomes (eBLT) were used for on-bead fragmentation and adapter ligation, followed by limited-cycle PCR to incorporate unique dual indexes. A double-sided size selection and cleanup with Illumina Purification Beads was performed to generate normalized pre-enrichment libraries.

### 2.6. Hybridization and Target Capture

Pre-enrichment libraries (~300–400 bp) were pooled (up to 12-plex) and hybridized with 120-mer biotinylated probes (Twist for Illumina Exome 2.5) (Twist for Illumina Exome 2.5) at 62 °C. Hybridized targets were captured using streptavidin magnetic beads, followed by washing, elution, and post-capture amplification.

### 2.7. Final Purification and Sequencing

Final bead-based cleanup was performed using Illumina Purification Beads. Library quality and fragment size distribution were verified using Agilent TapeStation and Qubit. Libraries were sequenced on an Illumina platform.

### 2.8. Data Analysis

VCF files were analyzed using the Franklin™ platform (Genoox, https://franklin.genoox.com) (accessed on 9 May 2026). Variant interpretation was performed in accordance with the American College of Medical Genetics and Genomics (ACMG) guidelines for the evaluation of pathogenicity.

## 3. Results

Of the 132 patients suspected of PH, mutations in the *AGXT* gene were identified by Sanger sequencing in 95 cases, corresponding to a diagnostic yield of 72%. The remaining 37 patients underwent targeted panel testing, which identified an *AGXT* variant in two additional patients. The 35 unresolved cases were subsequently analyzed by WES, yielding a definitive diagnosis in 6 additional patients, while the remaining 29 cases remained genetically unresolved.

These findings revealed a marked predominance of variants located in exons 7, 1, 2, and 10. The most frequently identified variant was the c.731T>C (p.Ile244Thr), detected in 126/264 alleles and observed in the homozygous state in 59 patients.

**Figure 1:**
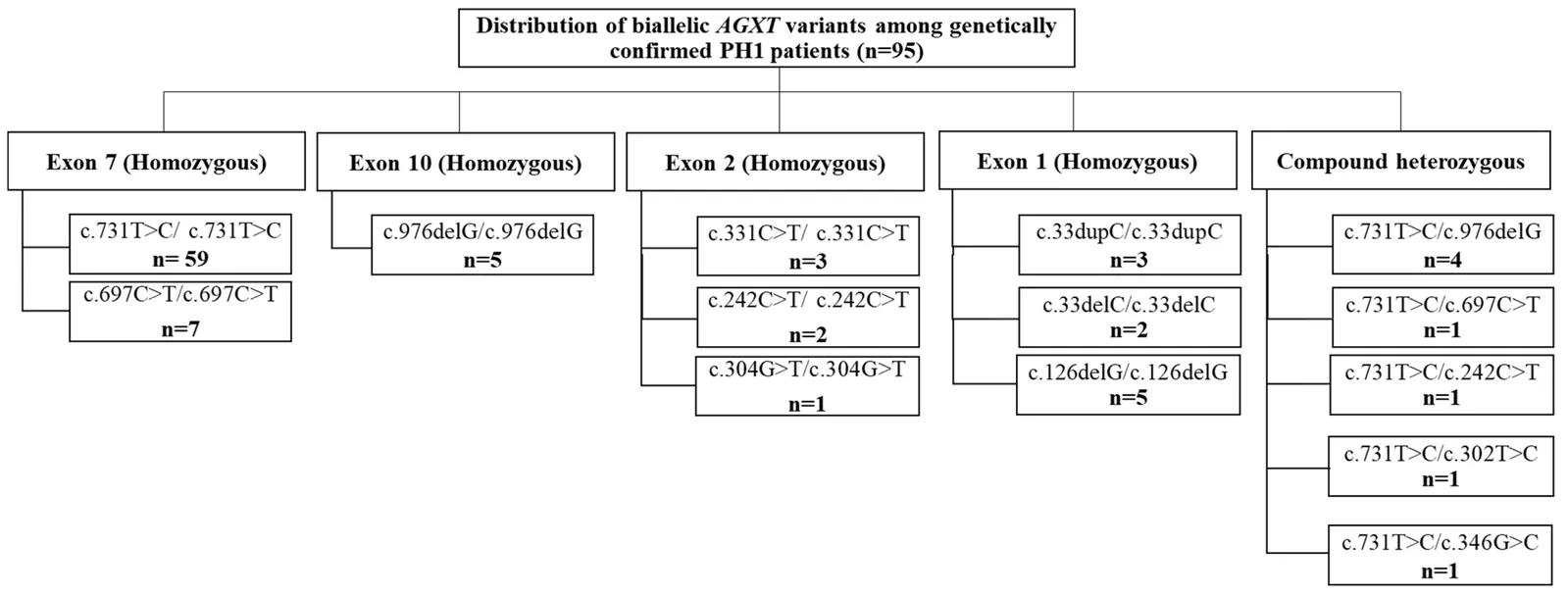
Distribution of biallelic *AGXT* variants identified among 95 patients with PH.

Another variant was detected in exon 7: the c.697C>T (p.Arg233Cys) was identified in 15/264 alleles, including 7 patients in the homozygous state. Additional variants were identified in exons 10, 2, and 1, including c.976delG, c.331C>T, c.242C>T, c.304G>T, c.33dupC, c.33delC, and c.126dupG (Figure 1).

Compound heterozygous variants were observed in 8 patients, all involving c.731T>C in combination with another *AGXT* variant, including c.976delG, c.697C>T, c.242C>T, c.302T>C, or c.346G>C. All variants had been previously reported and classified as pathogenic or likely pathogenic according to ACMG classification, except for c.346G>C. This novel variant was in line with the interpretation rule of “Pathogenic,” including the following criteria: PS1; PP3; PP5; PM1; PM5, and PM2.

In addition to variants detected by targeted Sanger sequencing of these 4 exons, two further variants, c.603C>A in exon 6 and c.919del in exon 9 of *AGXT*, were identified in the homozygous state by targeted panel. The distribution of point variants in the *AGXT* gene is summarized in Table 1.

Three heterozygous variants classified as variants of unknown significance (VUS) were detected by the targeted panel in three different patients: c.31C>G (p.Pro11Ala) in the *AGXT* gene, and c.491T>C (p.Ile164Thr) and c.374G>A (p.Arg125Gln) in the *GRHPR* gene. No second variant was identified in trans in these cases.

WES was carried out in 35 patients and revealed variants in other genes: *SI*, *SLC5A1*, *CLCN5*, *ATP6V0A4*, and *SLC4A1* in 6 patients. Detailed results, including variant classification, zygosity, and clinical interpretation, are summarized in Table 2.

## 4. Discussion

The present study is the largest molecular investigation of primary hyperoxaluria performed in a Moroccan cohort, including 132 patients clinically suspected of PH, in order to provide an overview of the mutational spectrum in this population. The cohort showed a predominance of pediatric-onset disease, with a median age at onset of 4 years and a mean age at onset of 7.7 ± 9.8 years but with a very wide range from the neonatal period to adulthood (42 years). The mean age at diagnosis was 8.5 years. Although the interval between symptom onset and diagnosis was relatively short in some patients, delayed diagnosis remains an important issue in Morocco, as well as in other developing countries [8]. This delay is largely attributable to the limited access to metabolic testing and the absence of urinary glycolate measurement. The clinical presentation in this cohort is associated with nephrolithiasis, nephrocalcinosis, elevated urinary oxalate and progression to ESRD. Early genetic testing, especially in patients with recurrent nephrolithiasis or positive family history, remains essential to improve patient management and prognosis.

### 4.1. Recurrent Molecular Findings in Sanger Sequencing Results

Our results highlight the predominance of the *AGXT* biallelic variants in 95 patients, due to the recurrence of pathogenic variants clustered in exons 1, 2, 7, and 10, particularly the founder variant c.731T>C (p.Ile244Thr). The mutational profile was dominated by c.731T>C (p.Ile244Thr), identified in 126/264 alleles and found in the homozygous state in 59 patients.

To our knowledge, Boualla et al. were the first to report the high frequency of the c.731T>C (p.Ile244Thr) variant in Moroccan patients, with a frequency of 84%. The same study estimated the carrier frequency of the *AGXT* recurrent mutation p.Ile244Thr in Morocco at approximately 1.6%, and the prevalence of the PH1 mutation among Moroccans was then estimated to range from 1/7267 to 1/6264 [3]. Remarkably, our cohort shows a high rate of consanguinity and a positive family history, highlighting the recurrence of this variant and supporting its contribution to the autosomal recessive inheritance pattern of PH1. These findings confirm the existence of mutational hotspots in the Moroccan population and support the establishment of a simplified, exon-focused molecular diagnostic strategy adapted to the local context. The high prevalence of the c.731T>C variant observed in this study aligns with findings from other North African and Spanish populations, particularly Tunisia and Egypt, where the same mutation has been reported with comparable frequencies. This recurrence confirms the founder effect of this mutation across the Mediterranean basin [3,9,10,11]. This variant, which causes the substitution of isoleucine by threonine at position 244, is associated with variable clinical severity, from childhood nephrolithiasis and nephrocalcinosis to renal failure. In addition, many studies confirm that missense variants in *AGXT* cause PH1 by disrupting AGT enzyme folding, stability, dimerization, and peroxisomal targeting.

The second most common variant found was the c.697C>T (p.Arg233Cys) (7.9%), also located in exon 7 of *AGXT* gene. It was detected in six patients in the homozygous state and in one patient in compound heterozygous state with the recurrent I244T mutation (15/264 alleles). Although the pathogenicity of this variant has not yet been confirmed by functional studies, it is classified as pathogenic according to ACMG criteria.

Beyond these two variants, c.731T>C and c.697C>T, the study identified several recurrent pathogenic variants, including c.976delG in exon 10, c.331C>T, c.242C>T, c.304G>T in exon 2, c.33dupC, c.33delC, and c.126delG in exon 1. Most of these variants were found in the homozygous state, whereas compound heterozygous genotypes were less frequent and always carried c.731T>C with another *AGXT* variant (Figure 1).

In addition, the novel NM_000030.3 (*AGXT*): c.346G>C (p.Gly116Arg) variant was classified as pathogenic according to ACMG criteria, based on PS1, PM1, PM2, PM5, PP3, and PP5. Notably, another nucleotide substitution affecting the same cDNA position, NM_000030.3 (*AGXT*): c.346G>A, results in the same amino acid change, p.Gly116Arg, and has previously been reported as pathogenic, supporting the pathogenicity of this residue and variant effect. Although no functional validation or segregation analysis was available, the cumulative evidence was sufficient to support a pathogenic classification.

Our data support the implementation of a stepwise molecular approach tailored to the Moroccan population, in which Sanger sequencing of exons 7, 10, 2, and 1—particularly targeting the recurrent c.731T>C variant—could serve as a rapid and cost-effective first-line test.

### 4.2. Molecular Findings in Targeted Panel Results

The remaining 37 Sanger-negative patients subsequently underwent a targeted panel of the remaining exons of the *AGXT*, *GRHPR*, and *HOGA1* genes to detect rare or novel variants. This approach allowed us to identify additional *AGXT* variants, including c.603C>A in exon 6 and c.919del in exon 9 in the homozygous state. Similar distributions have been reported in highly consanguineous populations such as Saudi Arabia, where PH1 accounted for 56% of cases, followed by PH2 (GRHPR) in 16% of cases [12].

Moreover, three heterozygous variants classified as VUS were detected in three different patients: c.31C>G (p.Pro11Ala) in the *AGXT* gene, and c.491T>C (p.Ile164Thr) and c.374G>A (p.Arg125Gln) in the *GRHPR* gene. No second variant was identified in trans in these cases. Nevertheless, a missed second variant cannot be fully excluded because targeted sequencing may fail to detect deep intronic variants, regulatory changes, complex rearrangements, or some copy number variants (CNVs). However, this panel showed a limited diagnostic contribution, providing a conclusive diagnosis in only 2 patients.

### 4.3. Other Genetic Findings and Diagnostic Reassessment

The 35 unresolved cases were therefore further investigated by WES, which revealed variants in other genes (including *SI*, *SLC5A1*, *CLCN5*, *ATP6V0A4*, and *SLC4A1*) in six patients (Table 2). These findings are important because several inherited disorders may present with overlapping renal phenotypes, including nephrolithiasis and nephrocalcinosis. Nephrolithiasis and nephrocalcinosis were common referral features in this cohort of patients suspected of PH; however, these manifestations are non-specific and require biochemical and/or molecular confirmation. This suggests that patients initially referred to as “PH-suspected” may actually have an alternative genetic disorder with clinical features overlapping with PH. Similar observations have been reported in pediatric NL/NC cohorts, where genetic testing enabled the identification of heterogeneous monogenic etiologies, including distal renal tubular acidosis, Dent disease, and primary hyperoxaluria [13].

The hemizygous *CLCN5*: c.925C>T (p.Arg309Cys) variant, classified as a VUS, should be interpreted in the context of Dent disease type 1, an X-linked tubulopathy associated with hypercalciuria, nephrolithiasis, nephrocalcinosis, and renal impairment. Thus, *CLCN5* represents an important differential diagnosis in patients with PH-like renal manifestations rather than a primary hyperoxaluria gene [13,14].

Identification of pathogenic variants in *ATP6V0A4*: c.2308C>T (p.Arg770Ter) and *SLC4A1*: c.1765C>T (p.Arg589Cys) is particularly important. These genes are implicated in inherited renal tubular disorders that may overlap with PH1 clinically due to the presence of nephrolithiasis and nephrocalcinosis [15,16].

Variants identified in ATP6V0A4, SLC4A1, and CLCN5 were not considered causative of primary hyperoxaluria. These findings represent alternative molecular diagnoses in patients initially referred for evaluation of suspected PH on the basis of non-specific clinical manifestations.

In our cohort, two homozygous variants in the SI gene were identified. The splice site variant c.4840_4841+2del was previously reported in ClinVar and classified as Pathogenic (SCV002267603.5). Loss-of-function variants in SI are known to be pathogenic. The second variant, c.2105T>C, is a novel variant classified according to ACMG as a VUS. The amino acid substitution is probably located in the isomaltase region. *SI* variants are responsible for congenital sucrase-isomaltase deficiency characterized by chronic osmotic diarrhea and gastrointestinal symptoms. *SI* deficiency has rarely been linked to kidney stones or NC, probably through secondary mechanisms such as dehydration and altered intestinal calcium oxalate handling [17,18]. Belmont et al. described nephrocalcinosis as a rare complication of congenital sucrase-isomaltase deficiency, suggesting that SI-related malabsorption may contribute to a secondary lithogenic phenotype [17].

Similarly, a homozygous variant in *SLC5A1*: c.1673G>A (p.Arg588His), a gene associated with congenital glucose–galactose malabsorption, was identified. Recently, nephrocalcinosis has been reported as a rare complication of *SLC5A1*-related glucose–galactose malabsorption, probably secondary to chronic diarrhea, dehydration, and metabolic imbalance [19]. Variants in *SI* and *SLC5A1* highlight the relevance of intestinal malabsorption disorders as differential diagnoses in patients with nephrocalcinosis or stone disease, although these genes are not classical PH genes. Moreover, after a reverse phenotyping, we noted that these patients with SI and SLC5A1 variants have consistent clinical signs such as diarrhea, abdominal pain and growth retardation.

Nevertheless, this study has several limitations. The absence of biochemical confirmation (urinary oxalate and glycolate quantification) represents a constraint in Morocco. The presence of nephrocalcinosis or recurrent stones should not automatically lead to a diagnosis of PH, especially when biallelic *AGXT* variants are absent or when the biochemical profile is atypical. Nephrolithiasis and nephrocalcinosis were common referral features in this cohort of patients suspected of PH; however, these manifestations are non-specific and require biochemical and/or molecular confirmation. Another major limitation of this retrospective study is the lack of biochemical data in some patients. This limited diagnostic certainty in unresolved and monoallelic cases, which were consequently not classified as molecularly confirmed PH cases. In resource-limited settings, specialized biochemical assays such as accurate 24-h urinary oxalate quantification or plasma oxalate measurement may be unavailable, incomplete, or logistically challenging. In this context, molecular analysis represents a valuable complementary diagnostic tool in patients with a strong clinical suspicion of PH, particularly when biochemical confirmation is lacking or difficult to obtain. By enabling molecular confirmation and identifying alternative genetic diagnoses, targeted gene panels and WES can help reduce diagnostic uncertainty and guide appropriate clinical management.

Overall, first-line Sanger sequencing identified biallelic pathogenic or likely pathogenic *AGXT* variants in 95/132 patients suspected of PH, corresponding to a diagnostic yield of 72%. Additional targeted PH panel sequencing and WES increased the final cumulative diagnostic yield to 103/132 patients (78%), while 29/132 patients (22%) belong to the gray area of undefined PH [20,21,22]. These findings suggest the existence of additional, not yet identified, genetic defects in glyoxylate metabolism and, importantly, indicate that the prevalence of PH may be higher than currently estimated.

**Figure 2:**
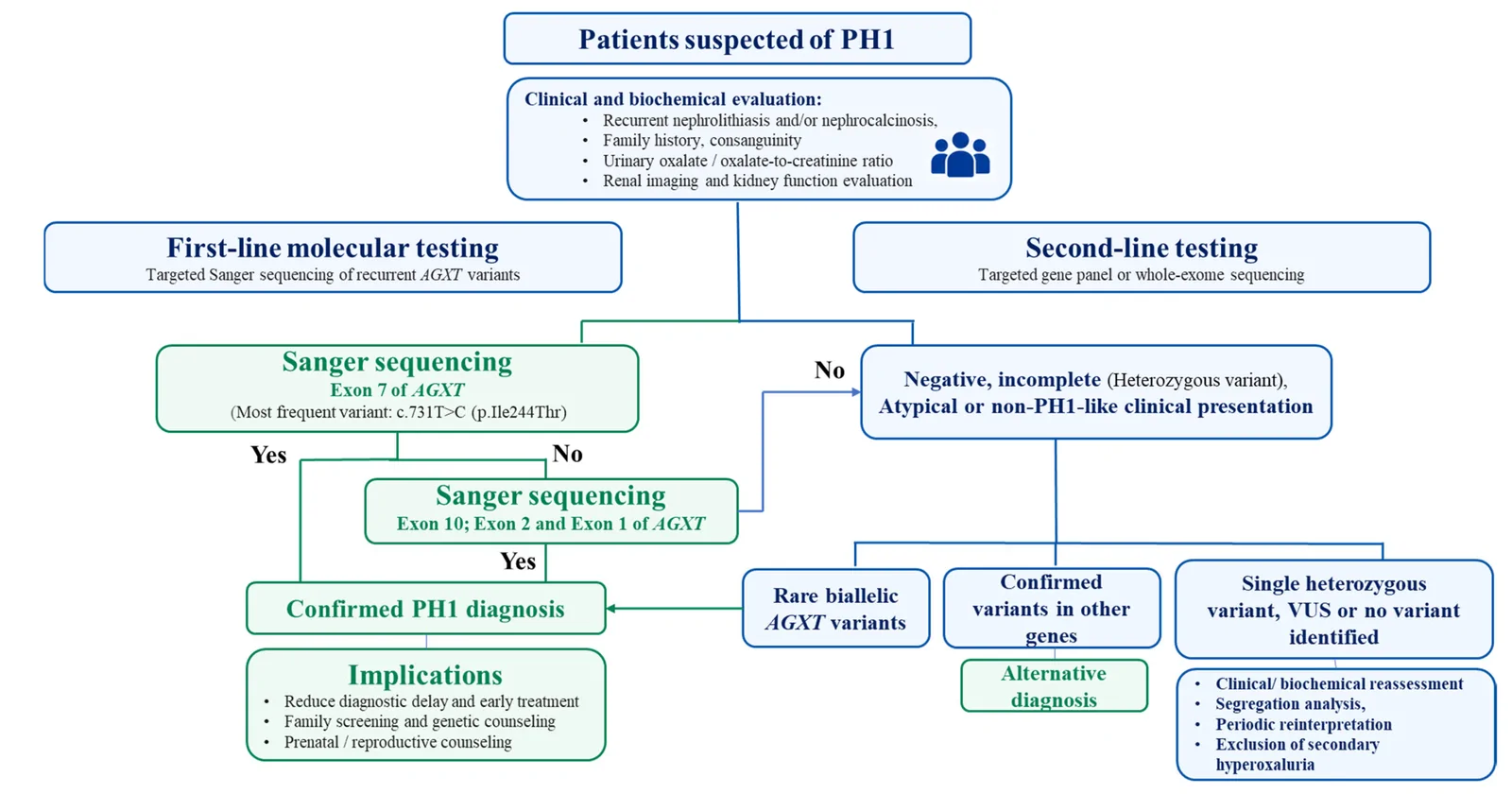
Proposed diagnostic strategy for suspected primary hyperoxaluria in Moroccan patients.

The identification of mutational hotspots and the development of an exon-focused screening strategy constitute major steps toward the optimization of molecular diagnosis in resource-limited settings. Moreover, the integration of NGS in unresolved cases broadens the diagnostic scope and highlights that PH-like presentations may result from alternative genetic etiologies (Figure 2). Our strategy may reduce diagnostic delay, enable early therapeutic intervention, facilitate genetic counseling, and contribute to better prevention of severe renal outcomes in affected families.

## 5. Conclusions

In conclusion, this study provides the largest molecular characterization of PH-suspected patients in Morocco and underscores the *AGXT*: c.731T>C variant as a recurrent mutation in the Moroccan population. Genetic testing has important therapeutic implications in PH, as it can identify patients who may benefit from pyridoxine therapy and support access to targeted treatments such as RNA interference drugs. Therefore, molecular diagnosis is essential not only for confirming PH, but also for guiding personalized treatment, prognosis assessment, and long-term clinical management. Finally, the stepwise workflow described herein allows reliable molecular diagnosis of PH in the Moroccan context, supporting early and accurate diagnosis of affected patients even without prior clinical subtyping.

## Figures and Tables

**Table 1:** *AGXT* variants identified by targeted Sanger sequencing and targeted panel sequencing in Moroccan patients with PH1.

Gene	Variant (cDNA)	Protein Change	Location	Allele Frequency	Prediction (ACMG)	N/R	Detection Method
*AGXT* *n* = 132 (264 alleles)	c.731T>C	p.Ile244Thr	Exon 7	126/264	Pathogenic (PP3; PP5; PM1; PM5; PS3; PM2)	R	Sanger
	c.697C>T	p.Arg233Cys	Exon 7	15/264	Pathogenic (PS3; PP3; PM1; PM2; PM5)	R	Sanger
	c.976delG	p.Val326TyrfsTer15	Exon 10	14/264	Pathogenic (PVS1; PP5; PM2)	R	Sanger
	c.331C>T	p.Arg111Ter	Exon 2	6/264	Pathogenic (PVS1; PP5; PM2)	R	Sanger
	c.242C>T	p.Ser81Leu	Exon 2	5/264	Pathogenic (PP3; PP5; PM1; PM5; PM2)	R	Sanger
	c.304G>T	p.Val102Phe	Exon 2	2/264	Likely Pathogenic (PP3; PM1; PM5; PM2)	R	Sanger
	c.302T>C	p.Leu101Pro	Exon 2	1/264	Pathogenic (PP3; PP5; PM1; PM2)	R	Sanger
	c.346G>C	p.Gly116Arg	Exon 2	1/264	Pathogenic (PS1; PP3; PP5; PM1; PM5; PM2)	N	Sanger
	c.33delC	p.Lys12ArgfsTer34	Exon 1	4/264	Pathogenic (PVS1; PP5; PM2)	R	Sanger
	c.33dupC	p.Lys12Glnfs Ter156	Exon 1	6/264	Pathogenic (PVS1; PP5; PM2)	R	Sanger
	c.126dupG	p.Glu137ThrfsTer77	Exon 1	10/264	Pathogenic (PVS1; PP5; PM2)	R	Sanger
	c.603C>A	p.Asp201Glu	Exon 6	2/264	Pathogenic (PP5; PM1; PM5; PM2; PP3)	R	Targeted Panel
	c.919del	p.L307CfsTer 5	Exon 9	2/264	Likely Pathogenic (PVS1; PM2)	R	Targeted Panel

R, Reported; N, Novel; ACMG, American College of Medical Genetics and Genomics.

**Table 2:** Summary of other variants identified by targeted panel and WES.

Gene	Inheritance	Variant (cDNA)	Protein Change	Zygosity	N/R	Prediction (ACMG)	Number of Cases	Detection Method	Clinical Comment
*SI*	AR	c.4840_4841+2del	p.?	Homozygous	R	Pathogenic (PVS1; PP5; PM2)	1	WES	Sucrase-isomaltase deficiency OMIM#222900
		c.2105T>C	p.Leu702Pro	Homozygous	N	VUS (PP3; PM2)	1	WES	
*CLCN5*	XLR	c.925C>T	p.Arg309Cys	Hemizygous	R	VUS (PP2; PP3)	1	WES	Nephrolithiasis, type I OMIM#310468
*ATP6V0A4*	AR	c.2308C>T	p.Arg770Ter	Homozygous	R	Pathogenic (PVS1; PP5; PM2)	1	WES	Distal renal tubular acidosis 3 OMIM#602722
*SLC4A1*	AD	c.1765C>T	p.Arg589Cys	Heterozygous	R	Pathogenic (PP5; PM1; PM5; PM2)	1	WES	Distal renal tubular acidosis 1 OMIM#179800
*SLC5A1*	AR	c.1673G>A	p.Arg588His	Homozygous	R	Likely pathogenic (PP5; PM2; PP2)	1	WES	Glucose/galactose malabsorption OMIM#606824
*GRHPR*	AR	c.491T>C	p.Ile164Thr	Heterozygous	N	VUS (PP2; PM2)	1	Targeted Panel	Hyperoxaluria, primary, type II OMIM#260000
		c.374G>A	p.Arg125Gln	Heterozygous	N	VUS (PP2; PM2)	1	Targeted Panel	
*AGXT*	AR	c.31C>G	p.Pro11Ala	Heterozygous	R	VUS (PM5; PM2; PP2)	1	Sanger	Hyperoxaluria, primary, type 1 OMIM#259900

AD, Autosomal Dominant; AR, Autosomal Recessive; XLR, X-linked recessive; N, Novel; R, Reported; ACMG, American College of Medical Genetics and Genomics; VUS, Variant of Unknown significance; WES, Whole-Exome sequencing.

## Data Availability

The data presented in this study are available on request from the corresponding author. The data are not publicly available due to privacy and ethical restrictions related to the confidentiality of patients’ clinical and genetic information.

## References

[1] Groothoff J.W., Metry E., Deesker L., Garrelfs S., Acquaviva C., Almardini R., Beck B.B., Boyer O., Cerkauskiene R., Ferraro P.M. (2023). Clinical practice recommendations for primary hyperoxaluria: An expert consensus statement from ERKNet and OxalEurope. Nat. Rev. Nephrol..

[2] Cochat P., Rumsby G. (2013). Primary hyperoxaluria. N. Engl. J. Med..

[3] Boualla L., Tajir M., Oulahiane N., Lyahyai J., Laarabi F.Z., Chafai Elalaoui S., Soulami K., Ait Ouamar H., Sefiani A. (2015). AGXT Gene Mutations and Prevalence of Primary Hyperoxaluria Type 1 in Moroccan Population. Genet. Test. Mol. Biomark..

[4] Jaouad I.C., Elalaoui S.C., Sbiti A., Elkerh F., Belmahi L., Sefiani A. (2009). Consanguineous marriages in Morocco and the consequence for the incidence of autosomal recessive disorders. J. Biosoc. Sci..

[5] Rumsby G., Williams E., Coulter-Mackie M. (2004). Evaluation of mutation screening as a first line test for the diagnosis of the primary hyperoxalurias. Kidney Int..

[6] Purdue P.E., Lumb M.J., Fox M., Griffo G., Hamon-Benais C., Povey S., Danpure C.J. (1991). Characterization and chromosomal mapping of a genomic clone encoding human alanine: Glyoxylate aminotransferase. Genomics.

[7] Beck B.B., Hoyer-Kuhn H., Göbel H., Habbig S., Hoppe B. (2013). Hyperoxaluria and systemic oxalosis: An update on current therapy and future directions. Expert Opin. Investig. Drugs.

[8] Soliman N.A., Mabrouk S. (2022). Primary hyperoxaluria type 1 in developing countries: Novel challenges in a new therapeutic era. Clin. Kidney J..

[9] Al Riyami M.S., Al Ghaithi B., Al Hashmi N., Al Kalbani N. (2015). Primary hyperoxaluria type 1 in 18 children: Genotyping and outcome. Int. J. Nephrol..

[10] Nagara M., Tiar A., Halim N., Rhouma F., Messaoud O., Bouyacoub Y., Kefi R., Hassayoun S., Noura Z., Ammar M. (2013). Mutation spectrum of primary hyperoxaluria type 1 in Tunisia: Implication for diagnosis in North Africa. Gene.

[11] Rhuma N., Fituri O., Sabei L. (2018). Mutational analysis of AGXT gene in Libyan children with primary hyperoxaluria type 1 at Tripoli Children Hospital. Saudi J. Kidney Dis. Transplant..

[12] Alfadhel M., Umair M., Alghamdi M.A., Al Fakeeh K., Al Qahtani A.T., Farahat A., Shalaby M.A., Kari J.A., Raina R., Cochat P. (2023). Clinical and molecular characterization of a large primary hyperoxaluria cohort from Saudi Arabia: A retrospective study. Pediatr. Nephrol..

[13] Huang L., Qi C., Zhu G., Ding J., Yuan L., Sun J., He X., Wang X. (2022). Genetic testing enables a precision medicine approach for nephrolithiasis and nephrocalcinosis in pediatrics: A single-center cohort. Mol. Genet. Genom..

[14] Lieske J.C., Milliner D.S., Beara-Lasic L., Harris P., Cogal A., Abrash E., Adam M.P., Bick S., Mirzaa G.M., Pagon R.A., Wallace S.E., Amemiya A. (1993). Dent Disease. GeneReviews^®^.

[15] Cogal A., Arroyo J., Shah R., Reese K., Walton B., Reynolds L., Kennedy G., Seide B., Senum S., Baum M. (2021). Comprehensive Genetic Analysis Reveals Complexity of Monogenic Urinary Stone Disease. Kidney Int. Rep..

[16] Trepiccione F., Walsh S.B., Ariceta G., Boyer O., Emma F., Camilla R., Ferraro P.M., Haffner D., Konrad M., Levtchenko E. (2021). Distal renal tubular acidosis: ERKNet/ESPN clinical practice points. Nephrol. Dial. Transplant..

[17] Belmont J.W., Reid B., Taylor W., Baker S.S., Moore W.H., Morriss M.C., Podrebarac S.M., Glass N., Schwartz I.D. (2002). Congenital sucrase-isomaltase deficiency presenting with failure to thrive, hypercalcemia, and nephrocalcinosis. BMC Pediatr..

[18] Sander P., Alfalah M., Keiser M., Korponay-Szabo I., Kovács J.B., Leeb T., Naim H.Y. (2006). Novel mutations in the human sucrase-isomaltase gene (SI) that cause congenital carbohydrate malabsorption. Hum. Mutat..

[19] Goel M., Suthar R., Dawman L. (2025). Congenital Glucose–Galactose Malabsorption Presenting as Hypertriglyceridemia and Medullary Nephrocalcinosis. Pediatr. Rep..

[20] Cochat P., Hulton S.-A., Acquaviva C., Danpure C.J., Daudon M., De Marchi M., Fargue S., Groothoff J., Harambat J., Hoppe B. (2012). Primary hyperoxaluria Type 1: Indications for screening and guidance for diagnosis and treatment. Nephrol. Dial. Transplant..

[21] Hopp K., Cogal A.G., Bergstralh E.J., Seide B.M., Olson J.B., Meek A.M., Lieske J.C., Milliner D.S., Harris P.C. (2015). Rare Kidney Stone Consortium. Phenotype-Genotype Correlations and Estimated Carrier Frequencies of Primary Hyperoxaluria. J. Am. Soc. Nephrol..

[22] Zhu X., Cheung W.W., Zhang A., Ding G. (2024). Mutation Characteristics of Primary Hyperoxaluria in the Chinese Population and Current International Diagnosis and Treatment Status. Kidney Dis..

